# Evaluation of the Expression of Programmed Death-Ligand 1 and Its Role in Differentiating Low-Grade and High-Grade Urothelial Carcinoma

**DOI:** 10.7759/cureus.62567

**Published:** 2024-06-17

**Authors:** Anushweta Singh, Mamta Kumari, Debaditya Haldar, Roushni Kumari, Nikhil Ranjan, Rajnikant Prasad

**Affiliations:** 1 Pathology, Indira Gandhi Institute of Medical Sciences (IGIMS) Patna, Patna, IND; 2 Urology, Indira Gandhi Institute of Medical Sciences (IGIMS) Patna, Patna, IND

**Keywords:** urothelial carcinoma, pd-l1, papillary, muscle invasive, bladder tumor

## Abstract

Background: Urothelial carcinoma (UC) is a common malignancy, predominantly affecting males. Many tumor cells use the interaction between programmed death-ligand 1 (PD-L1) and programmed death receptor (PD-1) to inactivate T-cells in the microenvironment and evade host immune response. Our study aims to evaluate the expression of PD-L1 in UC and correlate its expression with histomorphological parameters.

Materials and methods: After obtaining approval from the Institute Ethics Committee, we conducted a prospective observational study on transurethral resection of urinary bladder tumor (TURBT) and cystectomy specimens histopathologically diagnosed as UC between 2022 and 2023, comprising 50 cases. All standard protocol was followed and immunohistochemistry (IHC) was done using PD-L1 with rabbit anti-human PD-L1 monoclonal antibody (Clone: IHC411; Biogenics Inc., San Francisco, CA, USA).

Results: Among the 50 cases of UC, the majority were papillary type (35 cases), high grade (28 cases), and non-muscle invasive (30 cases). Among the cases studied, 15 of them showed PD-L1 positivity; 55% of the cases of muscle-invasive bladder cancer were found to be positive for PD-L1 out of which the results were statistically significant.

Conclusion: PD-L1 expression by IHC staining can differentiate between muscle-invasive and non-muscle-invasive UC cases. This observation allows for further exploring the potential role of immune checkpoint inhibitors in adjuvant and neoadjuvant therapy, especially in muscle-invasive cases of UC.

## Introduction

Urothelial carcinoma (UC) is the sixth most common carcinoma among males and 17th most common among females, based on International Agency for Research on Cancer (IARC) data [1]. Lower urinary tract, including urinary bladder (UB) and urethra, constitute 90-95% of UC cases. As per GLOBOCAN 2020, UB cancer is the 17th most common malignancy in India [2]. Genetic, environmental factors, schistosomiasis and pelvic irradiation are all implicated in the pathogenesis [3].

Among the existing treatment modalities, Bacillus-Calmette-Guerin (BCG) intravesical therapy is advocated for T1 tumors in UC. A new domain of immunotherapy has emerged which focuses on immune checkpoint inhibitors in UC. One such target is programmed death-ligand 1 (PD-L1) [4]. PD-L1 is a transmembrane protein belonging to the B7/CD28 co-stimulatory factor superfamily, which binds to the programmed death receptor (PD-1) [5]. PD-1 is expressed on cytotoxic T-cells and other immune cells, while PD-L1 is expressed on normal cells. Normal cells use this PD-1/PD-L1 interaction to inactivate the T-cells, thereby limiting damage to normal tissue. Many tumor cells upregulate PD-L1 expression to evade the body’s natural immune response. They use the PD-1/PD-L1 signaling just like normal cells to render the T-cells inactive, thereby escaping the immune cycle, and avoiding detection for elimination. The basis for using immunotherapeutic agents is to prevent this PD-1/PD-L1 interaction, thus keeping the immune system active, and preventing immunosuppression [6].

PD-L1 expression is seen in tissues like the placenta, pancreas, spleen, thymus and lymph nodes [7]. Immune checkpoint inhibitors have been used in the treatment of malignancies like non-small cell lung carcinoma, renal cell carcinoma, and melanoma among others, with an overall response rate of 16-100% [8]. Al Nabhani et al. observed a significant association between PD-L1 expression in UC and tumor grade in the Omani population, with higher expression in high-grade UC [9].

Since the controlling antigen is PD-L1 and the primary antibody used is a rabbit anti-human monoclonal antibody, the specificity and sensitivity of detecting PD-L1 in tissues were high. This improves the diagnostic process, impacts therapy choices throughout checkpoint inhibitor remedies, and offers dependable outcomes in each baseline medical and clinical analysis.

Our aim in this study is to explore the expression of PD-L1 in UC along with the correlation of PD-L1 score with histomorphological features. We have also attempted to assess the role of PD-L1 in differentiating low-grade from high-grade UC.

## Materials and methods

The Indira Gandhi Institute of Medical Sciences Patna Institutional Ethics Committee approval was obtained before conducting this study, with approval number 341/IEC/IGIMS/2021. All procedures performed in this study were in accordance with the Helsinki Declaration of 1975, as revised in 2000. Informed consent was obtained from all individual participants included in the study and confidentiality of the subjects was maintained.

This prospective hospital-based observational study included 50 patients who underwent transurethral resection of UB tumor (TURBT) or cystectomy in our hospital and histopathologically diagnosed as UC between 2022 and 2023. All UB tumors with diagnosis other than UC were excluded from our study.

Age and gender details were obtained from the Department of Urology. The TURBT and cystectomy specimens submitted to the Department of Pathology were fixed in 10% formalin solution and grossing was done according to standard protocol. Paraffin-embedded tissue blocks were prepared, sectioned and stained with routine Hematoxylin and Eosin (HE). The stained slides of tissue samples were microscopically analysed for various histomorphological parameters like histologic type of tumor, tumor grade, muscle invasion, lymphovascular invasion (LVI), and pathological T-staging. Tumor typing and grading was done according to the 2021 WHO classification of urothelial tumors. Staging was carried out as per the American Joint Committee on Cancer (AJCC) TNM system for staging of bladder cancer (8th edition) [10].

Immunohistochemistry (IHC) was done using PD-L1 to evaluate its expression and correlate with histomorphological findings in all the cases. Combined positive score (CPS) was calculated for all the cases of UC.

Immunohistochemistry

Cases diagnosed as UC on histomorphology were subjected to IHC analysis. Manual IHC staining was carried out on 5 μm thin paraffin-embedded tissue sections. Standard protocol was followed, and a pressure cooker was used for antigen retrieval. Endogenous peroxidase activity blocking was done using 3% hydrogen peroxide. Sections were incubated at four degrees Celsius overnight with primary antibody rabbit anti-human PD-L1 monoclonal antibody (Clone: IHC411; Biogenics Inc., San Francisco, CA, USA) diluted to 1:100. The next day after bringing them to room temperature and washing, the secondary antibody conjugated with horse radish peroxidase was applied and sections re-incubated for one hour. Chromogen (diaminobenzidine) was added, and counterstaining was done with Hematoxylin. For positive control, a tonsil specimen was used. Partial or complete light yellow to brown membranous staining in the tumor cells, and membranous with/without cytoplasmic staining in immune cells was considered as positive PD-L1 expression.

Interpretation of PD-L1 expression in UC

PD-L1 immunohistochemical expression was evaluated at 20x magnification for tumor cells (TC) as TC-Score and immune cells (IC) as IC-Score regardless of the staining intensity, and CPS was calculated in each case as per the guidelines mentioned in the 22C3 (pharmDx) interpretation manual (Agilent, Santa Clara, CA, USA) [6]. Partial or complete linear membranous staining of any intensity in the TC was considered as positive expression. IC included lymphocytes and macrophages while excluding plasma cells, neutrophils or eosinophils. Membranous and/or cytoplasmic staining of any intensity in the IC within the tumor nests and/or immediately adjacent stroma was considered as positive.

Percentage of TC showing positive staining allowed assignment of TC-Scores in Table 1. The scale ranges from 0 to 8, where a score of 0 means no staining or less than 1% of tumor cells stained. A score of 1 equates to 1.0% to less than 5.0% of the cancer cells exhibiting staining. A score of 2 ranges from 5% to less than 10% of tumor cells present with positive staining. A score of 2 is given to staining in 5% to less than 10%, whereas, a score of 3 is given to staining in 10% to less than 25%. A score of 4 is reached when staining is observed in 25%- <50% of the tumor cells. In the end, a score of 5 implies that tumor cells are positively stained and such cells are seen in greater than 50% of the tumor. In conclusion, there is the following six-point scale for evaluating the intensity of tumor cell staining: 0 - no staining; 1 - some cells stained weakly; 2 - some cells stained moderately; 3 - some cells stained darkly; 4 - more than 10% of cells stained darkly; 5 - at least 50% of a section of the tumor stained. The higher the score, the more the tumor cells were stained, which would indicate that the antigen or biomarker being targeted is expressed at a higher level in the tumor cells. Such a scoring system might be beneficial for the differential pathological analysis and reporting of immunohistochemistry outcomes.

**Table 1:** TC-Score Assignment Based on Percentage of TC Showing Positive Staining TC: tumor cells

TC-Score	Percentage of TC Showing Positive Staining
0	No staining to less than 1% cells
1	Staining in 1% to less than 5% cells
2	Staining in 5% to less than 10% cells
3	Staining in 10% to less than 25% cells
4	Staining in 25% to less than 50% cells
5	Staining in 50% or more cells

The percentage of IC showing positive staining was noted and IC-Scores were assigned in Table 2. Scoring system of the extent of positive staining per cell, where the percentage value is given. A score of 0 means that there is no staining or possibly up to 1% of cells stained. A score of 1 means 1% to <5% of the cells are stained out of all the high-power fields observed. A score of 2 means that less than 10% of the cells have positive staining but 5% of the cells are positive. Lastly, a score of 3 is assigned if the percentage of the cells that are positively stained is equal to 10% and above. This scoring system constitutes a set of guidelines for comparing the extent of the positive staining in a cell sample. The samples receive higher scores in the case in which a higher percentage of the cells show the stain, and the maximum possible total of 3 is assigned to the samples, in which at least 10% of the cells are stained. This makes it possible for several cell samples to be quantitatively graded and compared based on the observed positive staining.

**Table 2:** The IC-Scores based on the percentage of IC showing positive staining IC: immune cells

IC-Score	Percentage of Cells Showing Positive Staining
0	No staining to < 1% cells
1	1% to < 5% cells
2	5% to < 10% cells
3	≥ 10% cells

CPS was calculated in each case and defined as the number of cells expressing PD-L1 (TC + IC) divided by total number of viable TC (both PD-L1 staining and non-staining), and multiplied by 100. The result of the calculation may exceed 100, however, the maximum allowed CPS score was taken as 100.

The 22C3 (pharmDx) defines a CPS≥10 as positive PD-L1 expression in UC [6]. Theoretically, all tumor cells present in the tumor area (irrespective of PD-L1 staining status) should be included in the denominator. The total number of PD-L1 positive cells (TC + IC) in the tumor area form the numerator. This scoring system is cumbersome, and hence, the 22C3 manufacturer (Agilent) suggests selecting a portion of the tumor for scoring, which was followed in our study.

Statistical analysis was done using SPSS 22.0 software (IBM Corp., Armonk, NY, USA). Chi-square and Fisher’s exact tests were used for correlation. Results with p-value < 0.05 were considered statistically significant.

## Results

**Figure 1:**
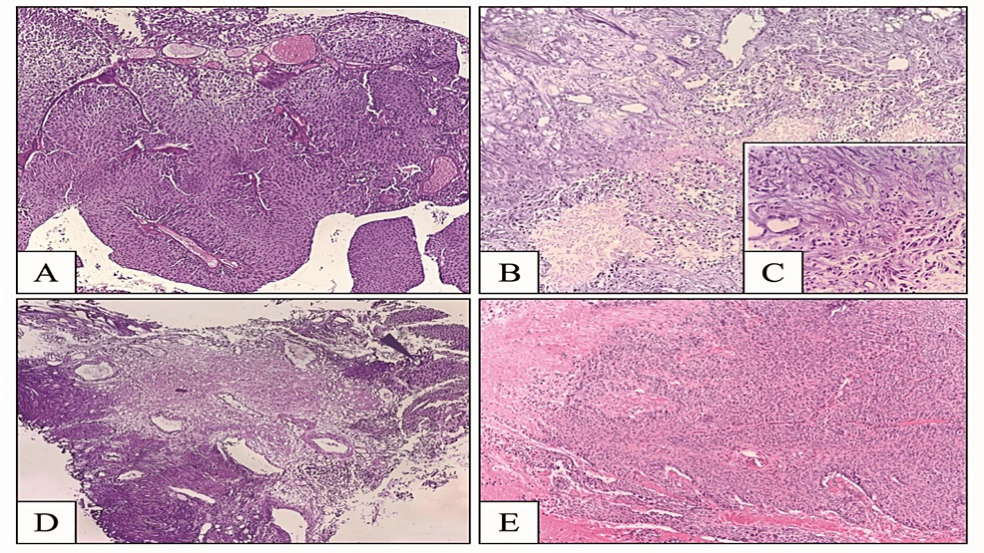
A – High-grade urothelial carcinoma, HE stains, 20x; B – Muscle invasive urothelial carcinoma, HE stain, 20x; C – Inset showing deep muscle invasion, HE stain, 40x; D – Arrowhead showing tumor cells infiltrating into muscle, HE stain, 10x; E – Urothelial carcinoma with squamous differentiation, HE stain, 20x HE: Hematoxylin and Eosin

Histopathological features and the status of PD-L1 expression in the 50 cases of UC have been summarised (Table 1), and it was found that 22 cases (44%) were low grade and the remaining 28 cases (56%) had high-grade UC (Figure 1A). Twenty cases (40%) were muscle invasive (Figure 1B-1D). Among the histologic types, papillary UC constituted the majority and accounted for 35 cases (70%), and the remaining included 10 cases of UC with lymphoepithelial variant and five cases of UC with squamous differentiation (Figure 1E).

**Figure 2:**
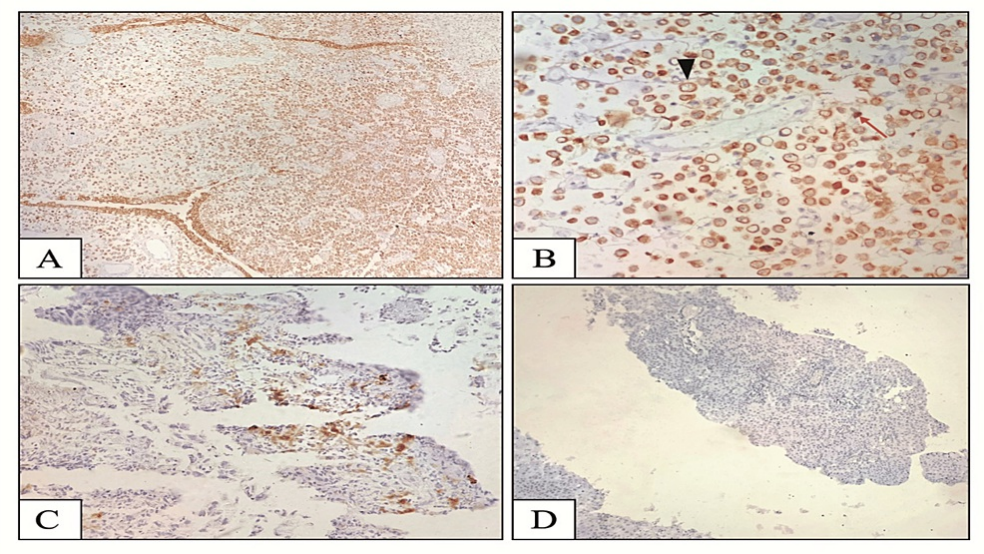
A – Tumor area showing PD-L1 positive expression, 10x; B – Black arrowhead showing PD-L1 positive staining on the cell membrane of tumor cells, red arrow showing PD-L1 positive cytoplasmic staining of immune cells, 20x; C – Immune cells showing PD-L1 positivity, 10x; D – PD-L1 negative expression, 10x PD-L1: programmed death-ligand 1

Among the 20 muscle-invasive cases, 11 cases (55%) showed PD-L1 positivity (CPS≥10) (Figure 2A-2C). Out of the 30 muscle non-invasive cases, PD-L1 was expressed in only four cases (13%) and the majority of non-muscle-invasive cases (87%) were negative for PD-L1 (CPS<10) (Figure 2D). This was a statistically significant finding. Also, TC-Score and IC-Score assigned were correlated with low-grade and high-grade UC (Table 2), but this was not statistically significant.

Assessment of immunohistochemical staining results of PD-L1

Positive PD-L1 expression was demonstrated as CPS≥10 in 15 cases (30%). The relation between the IHC staining results of PD-L1 expression and different clinicopathological parameters is summarized in Table 3. Among the 50 UC cases, 15 cases (30%) showed PD-L1 positive expression, which included 13 out of 35 infiltrating papillary UC cases (37%), one out of 10 cases of UC with lymphoepithelial variant (10%) and one out of five UC cases with squamous differentiation (20%).

**Table 3:** Correlation of PD-L1 expression with the clinical-histopathological features of UC p-value < 0.05 was significant PD-L1: programmed death-ligand 1, UC: urothelial carcinoma, CPS: combined positive score

Parameters	Category	Total no. of cases (% of Total cases)	No. of cases with positive PD-L1 expression, CPS≥10 (% of Total cases)	No. of cases with negative PD-L1 expression, CPS<10 (% of Total cases)	p-value
Age	> 60 yrs	30 (60%)	9 (30%)	21 (70%)	1.0
	< 60 yrs	20 (40%)	6 (30%)	14 (70%)	
Gender	Male	39 (78%)	12 (31%)	27 (69%)	0.9
	Female	11 (22%)	3 (27%)	08 (73%)	
Muscle invasion	Invasive	20 (40%)	11 (55%)	09 (45%)	0.001^*^
	Non-invasive	30 (60%)	04 (13%)	26 (87%)	
Histologic type	Papillary	35 (70%)	13 (37%)	22 (63%)	0.09
	Non-papillary	15 (30%)	02 (13%)	13 (87%)	
Histologic grade	Low grade	22 (44%)	05 (23%)	17 (77%)	0.32
	High grade	28 (56%)	10 (36%)	18 (64%)	
Lymphovascular invasion	Present	33 (66%)	10 (30%)	23 (70%)	0.95
	Absent	17 (34%)	05 (29%)	12 (71%)	
T-stage	T1	02 (17%)	01 (50%)	01 (50%)	1.0
	T2	07 (58%)	05 (71%)	02 (29%)	
	T3	02 (17%)	02 (100%)	0 (0%)	
	T4	01 (8%)	01 (100%)	0 (0%)	

We investigated 50 patients with bladder cancer, and the results showed that there was PD-L1 positivity in 30% of tumors with IHC CPS >10. Positive PD-L1 expression was identified in 55% of MI tumors and 13% of NMIs, and the difference was significant at p=0.001. There was no relationship between PD-L1 result and other clinicopathological variables, including the age, gender, structure of the tumor, its grade, lymph vascular invasion, and T stage, although the size of some categories in the T stage groups is small. The patient population was comprised of the majority of male patients (78%) aged more than 60 years (60%), with papillary histology (70%) and LVI (66%). In conclusion, this work reveals that muscle-invasive bladder cancer (MIBC) is 5% more likely to have PD-L1 positivity compared to non-muscle invasive disease. Further investigations are warranted with larger patient cohorts to elucidate the correlation between PD-L1 expression and clinicopathologic features.

Among the 20 muscle-invasive cases, 11 cases (55%) showed PD-L1 positivity (CPS≥10) (Figure 2A-2C). Out of the 30 muscle non-invasive cases, PD-L1 was expressed in only four cases (13%) and most non-muscle invasive cases (87%) were negative for PD-L1 (CPS<10) (Figure 2D). This was a statistically significant finding. Also, the TC-score and IC-score assigned were correlated with low-grade and high-grade UC in Table 4, but this was not statistically significant. Table 2 displays the data that indicate the difference between low-grade and high-grade urothelial carcinoma (UC) according to TC scores and IC scores. As such, most low-grade and high-grade UC cases were rated at a score of 0 for both scores. Especially, regarding the TC-score, 17 cases with low-grade UC were classified, and 18 cases with high-grade UC, which was given a score of 0. Likewise, the score of IC for the 17 low-grade UC cases and 18 high-grade UC cases was 0. The TC and IC scores distributed across the remaining score levels (1-5 and 1-3, respectively) contained one to three cases of low-grade UC and two to five cases of high-grade UC. The calculated p-value is 0.76, which suggests that there is no statistical association between low and high-grade UC cases about the TC-Score or IC-Score levels. Accordingly, the distribution of TC scores and IC scores for low-grade and high-grade UC is comparable.

**Table 4:** Correlation of TC-Score and IC-Score in low-grade and high-grade UC p-value < 0.05 was significant. TC: tumor cells, IC: immune cells, UC: urothelial carcinoma

Parameter	Score	Low-grade UC cases	High-grade UC cases	p-value
TC-Score	0	17	18	0.76
	1	01	02	
	2	03	05	
	3	01	03	
	4	00	00	
	5	00	00	
IC-Score	0	17	18	0.76
	1	01	02	
	2	03	05	
	3	01	03	

## Discussion

UC accounts for more than 90% of UB carcinoma and may be muscle invasive in 25% of cases, or superficial, that is, non-muscle invasive, being limited to the mucosa or lamina propria [11]. Precision therapy in the form of immune checkpoint inhibitors has been a major event in the management of advanced UB carcinoma. Few studies have explored PD-L1 expression in UC with pure or variant histology. Reis et al. demonstrated a higher rate of PD-L1 expression in UC with variant histology compared to pure UC [12]. Recently, the use of the individual or a panel of IHC markers has been suggested as a part of an integrated morpho-molecular approach [13].

Among the 50 cases of UC in our study, 15 cases (30%) showed positive PD-L1 expression (CPS≥10) which was higher than reports by Faraj et al., Boorjian et al., Inman et al., and Bellmunt et al., which showed 18%, 12.4%, 28% and 20% positivity respectively [14-17]. Our results were lower in comparison to those of Anand et al. and Zhang et al., where PD-L1 positivity was found to be 38% and 45% respectively [18,19]. These studies used either greater than or equal to 5% or 1% as the cut-off limit, but we have used CPS of greater than 10 as a cut-off limit. The discordant results are mainly due to the different reagents, clones of antibodies, different staining platforms, and different scoring algorithms used worldwide.

In our study, 13/35 cases (37%) of infiltrating papillary UC, one out of 10 cases (10%) of UC with lymphoepithelial variant, and one out of five cases (20%) of UC with squamous differentiation showed PD-L1 positivity. So, in our demographic area, the majority of UC cases received were those of infiltrating papillary UC, which showed a higher percentage of PD-L1 positivity than the non-papillary type, however, with no statistical significance. Our study showed male preponderance in PDL-1 positivity (31%) than females (27%) but also revealed no statistical significance in the above association. Most patients in our study belonged to the age group of 60 years and above (30 cases, 60%), and showed PD-L1 positive staining in nine out of 30 patients (30%) with no statistically significant p-value. This correlated with the findings of Inman et al. [16]. Faraj et al., however, revealed in their study that tumors from patients belonging to a younger age group showed higher PD-L1 positivity [14].

One eye-opening finding in our study was the significant association between muscle-invasive UC and PD-L1 positivity. Eleven of 20 cases (55%) of muscle-invasive UC showed PD-L1 positivity, and this was found to have a p-value of 0.001. This observed finding was against the findings of Inman et al., Bellmunt et al., and Nakanishi et al. [16,17,20]. Interestingly, Ding et al. concluded in a meta-analysis done in 2023 that expression of PD-L1 on tumor cells was associated with muscle-invasive UC. Around 4032 patients were included in the above-mentioned study [21]. There are a lot of ongoing studies exploring the utility of neoadjuvant anti-PD-1/PD-L1 agents on pathologic response rates in muscle-invasive UC. So, our study has the potential to become a cornerstone for the treatment of muscle-invasive UC patients with checkpoint inhibitor drugs like pembrolizumab in the future.

There was no significant association of the grade of the tumor with PD-L1 positivity (p-value 0.32) in UC. Likewise, LVI and pathological T-staging had no significant association with positive PD-L1 immunostaining. However, several studies have demonstrated an association with multiple prognostic indicators like the higher grade of tumor, increased resistance to BCG therapy, and muscle-invasive disease. TC-Score and IC-Score were also not statistically significant (p-value of 0.76 in both) when correlated with high-grade and low-grade UC. Works done by Boorjian et al., Xylinas et al. and Pichler et al. concluded that PD-L1 showed an effect on the prognosis and overall survival [15,22,23].

We are in the era of checkpoint inhibitor drugs like pembrolizumab, atezolizumab, etc. that target the PD-1/PD-L1 pathway in UC patients. Moreover, UC patients having a higher ratio of PD-L1 positive cells responded significantly to anti-PD-1/PD-L1 immunotherapy than those with lower ratios [24]. Although PD-L1 expression is observed on immune cells in patients receiving checkpoint inhibitors for advanced UC, more attention is being paid to the clinical relevance of PD-L1 expression on immune cells [25-29].

Muscle-invasive urothelial carcinoma and PD-L1 positivity were identified to be significantly connected in this research even though this opinion contradicts some earlier studies; however, it complies with the current meta-analysis findings. This suggests that the neoadjuvant anti-PD-1/PD-L1 agents may be useful in enhancing the pathologic outcomes of MIBC patients [30]. Since PD-L1 on immune cells is associated with immunotherapy treatment response in advanced urothelial carcinoma patients, more investigation is required on clinical uses for the biomarker and drug development involving the PD-1/PD-L1 pathway inhibitors involving pembrolizumab. In total, these results may be helpful for the development of further bladder cancer treatments.

Limitations

We used a manual method for IHC staining in our setup. This could be the reason for discrepant results in interpretation. Additional shortcomings included the lack of a validated specific antibody clone, and different reagents used. There is no universal standard or validated cut-off point for PD-L1 positivity, which resulted in varying observations between different studies. Our study was conducted over a limited period of one year and hence, only 50 patients of UC could be included. Also, pathological T-staging could be done for only 12 cases since the majority of specimens received were TURBT specimens.

## Conclusions

Assessing PD-L1 status in urothelial carcinoma and histomorphological characteristics analysis. In our study, we noted that out of the 50 initial cases, 15 (30%) had positive staining for PD-L1. Notably, there was a higher expression of PD-L1 in muscle-invasive tumors, with 55% of the cases being positive as opposed to only 13% in non-muscle-invasive tumors. This could also make PD-L1 even more critical in higher stages and aggressive cancer types. We found that using the scoring of PD-L1 immunohistochemistry, we can differentiate between muscle-invasive and non-muscle-invasive urothelial carcinoma. This has important clinical implications as regards the use of immune checkpoint inhibitors. It also further concerns the muscle-invasive cases in which higher positive PD-L1 staining indicates these patients might have more to gain from adjuvant or neoadjuvant immunotherapy targeting the PD-1/PD-L1 pathway. Taken together with the prior studies, our data further emphasized the prognostic and predictive significance of PD-L1 in urothelial carcinoma. Additional research needs to be done to explore the effectiveness of PD-L1 testing in terms of predictability and clinical relevance for immunotherapy application in this cancer type.

## References

[1] Bray F, Ferlay J, Soerjomataram I, Siegel RL, Torre LA, Jemal A (2018). Global cancer statistics 2018: GLOBOCAN estimates of incidence and mortality worldwide for 36 cancers in 185 countries. CA Cancer J Clin.

[2] Sung H, Ferlay J, Siegel RL, Laversanne M, Soerjomataram I, Jemal A, Bray F (2021). Global cancer statistics 2020: GLOBOCAN estimates of incidence and mortality worldwide for 36 cancers in 185 countries. CA Cancer J Clin.

[3] Epstein JI, Lotan TL (2015). The lower urinary tract and male genital system. Robbins & Cotran Pathologic Basis of Diseases, 9th edn.

[4] Martin-Liberal J, Ochoa de Olza M, Hierro C, Gros A, Rodon J, Tabernero J (2017). The expanding role of immunotherapy. Cancer Treat Rev.

[5] Keir ME, Butte MJ, Freeman GJ, Sharpe AH (2008). PD-1 and its ligands in tolerance and immunity. Annu Rev Immunol.

[6] PD-L1 IHC 22C3 pharmDx Interpretation Manual - Urothelial Carcinoma (2018). PD-L1 IHC 22C3 pharmDx Interpretation Manual - Urothelial Carcinoma. https://www.agilent.com/cs/library/usermanuals/public/29276_22C3_pharmdx_uc_interpretation_manual_us.pdf.

[7] Blank C, Gajewski TF, Mackensen A (2005). Interaction of PD-L1 on tumor cells with PD-1 on tumor-specific T cells as a mechanism of immune evasion: implications for tumor immunotherapy. Cancer Immunol Immunother.

[8] Ohaegbulam KC, Assal A, Lazar-Molnar E, Yao Y, Zang X (2015). Human cancer immunotherapy with antibodies to the PD-1 and PD-L1 pathway. Trends Mol Med.

[9] Al Nabhani S, Al Harthy A, Al Riyami M, Al Sinawi S, Al Rashdi A, Al Husseni S, Kumar S (2022). Programmed death-ligand 1 (PD-L1) expression in bladder cancer and its correlation with tumor grade, stage, and outcome. Oman Med J.

[10] Amin MB, Greene FL, Edge SB (2017). The Eighth Edition AJCC Cancer Staging Manual: continuing to build a bridge from a population-based to a more "personalized" approach to cancer staging. CA Cancer J Clin.

[11] de Jong JJ, Stoop H, Boormans JL, van Leenders GJ (2021). PD-L1 expression in urothelial bladder cancer varies more among specimen types than between companion assays. Virchows Arch.

[12] Reis H, Serrette R, Posada J (2019). PD-L1 expression in urothelial carcinoma with predominant or pure variant histology: concordance among 3 commonly used and commercially available antibodies. Am J Surg Pathol.

[13] Ravanini JN, Assato AK, Wakamatsu A, Alves VA (2021). Combined use of immunohistochemical markers of basal and luminal subtypes in urothelial carcinoma of the bladder: association with clinicopathological features and outcomes. Clinics (Sao Paulo).

[14] Faraj SF, Munari E, Guner G (2015). Assessment of tumoral PD-L1 expression and intratumoral CD8+ T cells in urothelial carcinoma. Urology.

[15] Boorjian SA, Sheinin Y, Crispen PL (2008). T-cell coregulatory molecule expression in urothelial cell carcinoma: clinicopathologic correlations and association with survival. Clin Cancer Res.

[16] Inman BA, Sebo TJ, Frigola X (2007). PD-L1 (B7-H1) expression by urothelial carcinoma of the bladder and BCG-induced granulomata: associations with localized stage progression. Cancer.

[17] Bellmunt J, Mullane SA, Werner L (2015). Association of PD-L1 expression on tumor-infiltrating mononuclear cells and overall survival in patients with urothelial carcinoma. Ann Oncol.

[18] Anand G, Kaur H, Bhasin TS (2022). Expression of PD-L1 in urothelial carcinoma and its association with clinicopathological parameters. Nat J Lab Med.

[19] Zhang J, Dickinson SI, Clark ND, Flaherty AL (2013). Expression of PD-L1 in primary urothelial carcinoma (UC). J Clin Oncol.

[20] Nakanishi J, Wada Y, Matsumoto K, Azuma M, Kikuchi K, Ueda S (2007). Overexpression of B7-H1 (PD-L1) significantly associates with tumor grade and postoperative prognosis in human urothelial cancers. Cancer Immunol Immunother.

[21] Ding X, Chen Q, Yang Z (2019). Clinicopathological and prognostic value of PD-L1 in urothelial carcinoma: a meta-analysis. Cancer Manag Res.

[22] Xylinas E, Robinson BD, Kluth LA (2014). Association of T-cell co-regulatory protein expression with clinical outcomes following radical cystectomy for urothelial carcinoma of the bladder. Eur J Surg Oncol.

[23] Pichler R, Heidegger I, Fritz J (2017). PD-L1 expression in bladder cancer and metastasis and its influence on oncologic outcome after cystectomy. Oncotarget.

[24] Liu J, Zhang C, Hu J (2018). Effectiveness of anti-PD-1/PD-L1 antibodies in urothelial carcinoma patients with different PD-L1 expression levels: a meta-analysis. Oncotarget.

[25] Canbey Goret C, Erkan M, Dogan M (2017). Signet-ring cell carcinoma metastasized into the meningioma: a case report. Int J Biol Pharm Sci.

[26] Rosenberg JE, Hoffman-Censits J, Powles T (2016). Atezolizumab in patients with locally advanced and metastatic urothelial carcinoma who have progressed following treatment with platinum-based chemotherapy: a single-arm, multicentre, phase 2 trial. Lancet.

[27] Panderi I, Perez K, Cao L (2017). Assessment of molecular differentiation in FFPE colon adenocarcinoma tissues using PCA analysis of MALDI IMS spectral data. J Appl Bioanal.

[28] Powles T, Eder JP, Fine GD (2014). MPDL3280A (anti-PD-L1) treatment leads to clinical activity in metastatic bladder cancer. Nature.

[29] Praveen M (2024). Multi-epitope-based vaccine designing against Junín virus glycoprotein: immunoinformatics approach. Futur J Pharm Sci.

[30] Shailaja AD, Pramodkumar JS (2023). Isolation, in-silico studies, and biological evaluation of higenamine from Annona squamosa L. against breast cancer. Int J Pharmaceut Qual Assur.

